# Investigation of causes of sex-related differences in ocular blood flow in healthy eyes determined by laser speckle flowgraphy

**DOI:** 10.1038/s41598-017-14118-0

**Published:** 2017-10-24

**Authors:** Takeshi Iwase, Kentaro Yamamoto, Kosei Yanagida, Eimei Ra, Yasuki Ito, Kenta Murotani, Hiroko Terasaki

**Affiliations:** 1 Department of Ophthalmology, Nagoya University Graduate School of Medicine, Nagoya, Japan; 2 Division of Biostatistics, Clinical Research Center, Aichi Medical University, Nagakute, Aichi Japan

## Abstract

Sex-related differences are present in the systemic and ocular blood flow. However, the cause of the sex-related differences has not been determined. We investigated the ocular blood flow, represented by the mean blur rate (MBR), on the optic nerve head (ONH) determined by laser speckle flowgraphy in 138 males (63.9 ± 8.9 years) and 194 females (63.5 ± 9.4 years). The correlations between the MBR on the ONH and the clinical data were determined. The overall ONH-MBR was significantly higher in females than males (*P* < 0.001). In addition, the levels of the hemoglobin and hematocrit were significantly lower in females than in males (*P* < 0.001). The ONH-MBR was negatively and significantly correlated with the levels of the hemoglobin and hematocrit (both, *P* < 0.001). Multiple regression analyses showed that the sex (β = 0.248, *P* < 0.001) was an independent factor correlated with the ONH-MBR when the clinical examination data were not included in the analyses. However, when the clinical examination data were included, the hemoglobin level (β = −0.295, *P* < 0.001) was an independent factor that contributed to the ONH-MBR but the sex was not. We conclude that the sex-related differences in the hemoglobin level and the negative correlation between hemoglobin and the ONH-MBR are the causes of the sex-related differences in the ONH-MBR.

## Introduction

Knowledge about the ocular blood flow is essential for understanding the pathological conditions associated with and the treatment of various ocular vascular diseases. The blood flow in the optic nerve head (ONH) has been reported to be reduced in some ocular diseases such as glaucoma^1–3^, retinitis pigmentosa^4,5^, and other vascular disorders. However, the pathogeneses of these blood flow-related ocular diseases have not been fully determined.

A variety of techniques have been developed to measure the retinal blood flow including fluorescein angiography^6^, radioactive microsphere technique^7^, and hydrogen clearance method^8^. These techniques are limited because of time intensiveness and poor reproducibility. More recently, Doppler optical coherence tomography (OCT)^9^, OCT angiography (OCTA)^10^, and optical microangiography (OMAG)^11,12^ have been developed, and they can measure the blood flow on the ONH and retina using high-resolution, depth-resolved imaging with high reproducibility. However, these techniques still have inherent limitations because the blood flow velocity is not measurable. Thus, it is important to find new ways to determine the blood flow more accurately, faster, and non-invasively to study these diseases.

Laser speckle flowgraphy (LSFG) is a promising candidate for such a method. LSFG is a non-invasive, real-time method that is used to measure the relative blood flow velocity in the ONH, retina, and choroid and one image can be collected in 4 seconds^13–15^. LSFG can detect the pattern of the speckle contrast produced by the interference of illuminating laser light scattered by the movement of erythrocytes in the blood vessels. It can measure the relative blood flow velocity as the mean blur rate (MBR) in the vessels^13–15^. The values obtained by LSFG have been shown to be significantly correlated with the blood flow values determined by the hydrogen gas clearance and by the microsphere methods^8,16^. These findings indicate that the values determined by LSFG are valid measures of the ocular blood flow and should be comparable among individuals. The analysis software can be used to calculate the MBRs in the blood vessels and tissues (capillaries) of the entire ONH, and the measurements have excellent reproducibility^17^ especially for the whole ONH^18^.

In comparing the blood flow among individuals, it is essential to consider the systemic constitution of the subjects. For example, it is known that there are sex-related differences in the systemic blood flow parameters^19–21^. Hayward *et al*. reported that the central arterial pressure waveforms in females differed significantly from those in males as assessed by carotid artery tonometry^20^. In the eye, Yanagida *et al*. reported that sex-related differences are present in the ocular blood flow in the ONH using LSFG. They concluded that the ONH-MBR was significantly higher in females than in males. In addition, five of the eight pulse waveform parameters determined by LSFG had significant sex-related differences.

It has been suggested that the sex-related differences in blood flow are due to inherent biological differences between males and females. For example, it is known that women tend to have lower body height and size, have higher heart rates, and lower cardiac outputs^22^. Some clinical examination findings are well known to have sex-related differences, e.g., hemoglobin concentration, hematocrit percentage, and serum creatinine levels. The hormonal differences, such as the presence of estrogen or testosterone, may also play a role in the sex-related differences in the blood flow between males and females^23,24^.

However, the cause of sex-related differences in the blood flow velocity has not been fully determined especially in the eye. One of the reasons for this absence is that systemic data including the clinical examinations are usually not obtained during the outpatient examinations in ophthalmic clinics.

The retinal vessels including those on the ONH can be observed noninvasively which allows clinicians to monitor the ocular circulatory system in healthy and diseased eyes. Investigations of the ocular blood flow can not only provide information on the ocular but also the systemic circulatory system. Thus, determining the mechanisms causing the sex-related differences in blood flow in the eye may also apply to the systemic circulatory system.

The purpose of this study was to determine what factors cause the sex-related differences in the ocular blood flow. To accomplish this, we evaluated the clinical findings, and calculated the correlations between the ONH-MBR determined by LSFG and the clinical examination findings in healthy subjects.

## Results

The demographic data of all of the subjects are shown in Table 1. One hundred thirty-eight males (mean age, 63.9 ± 8.9 years) and 194 females (mean age, 63.5 ± 9.4 years) were examined. The axial length (*P* < 0.001), systolic blood pressure (SBP; *P* = 0.001), diastolic blood pressure (DBP; *P* < 0.001), mean arterial blood pressure (MAP; *P* < 0.001), mean ocular perfusion pressure (MOPP; *P* < 0.001), erythrocyte count (*P* < 0.001), hemoglobin concentration (*P* < 0.001), hematocrit percentage (*P* < 0.001), white blood cell count (WBC; *P* < 0.001), triglycerides (*P* = 0.002), serum creatinine (*P* < 0.001), and body mass index (BMI; *P* = 0.006) were significantly higher in males than in females. The heart rate (*P* < 0.001), total cholesterol (*P* = 0.020), and high-density lipoprotein cholesterol (HDL; *P* < 0.001) were significantly higher in females than in males. No significant differences were observed between the sexes in the age, intraocular pressure (IOP), platelet count, HbA1c, TP, and low-density lipoprotein cholesterol (LDL).

**Table 1:** Clinical characteristics of all subjects.

Parameters	Male/Female	*P*-value
n	138/194	—
Age (years)	63.9 ± 8.9/63.5 ± 9.4	0.747
Intraocular pressure (mmHg)	13.5 ± 2.9/13.9 ± 2.7	0.205
Axial length (mm)	24.1 ± 1.1/23.7 ± 1.1	<0.001
Systolic blood pressure (mmHg)	136.4 ± 16.7/129 ± 12.7	<0.001
Diastolic blood pressure (mmHg)	82.9 ± 11.1/73.1 ± 12.2	<0.001
Mean arterial pressure (mmHg)	100.7 ± 12.0/91.9 ± 13.3	<0.001
Mean ocular perfusion pressure (mmHg)	53.7 ± 8.5/47.5 ± 8.9	<0.001
Heart rate (bpm)	68.7 ± 11.2/73.6 ± 11.9	<0.001
erythrocyte (×10^4^/µL)	473.8 ± 41.4/433.6 ± 35.1	<0.001
Hemoglobin (g/dL)	14.7 ± 11.1/13.1 ± 1.1	<0.001
Hematocrit (%)	44.8 ± 3.3/40.0 ± 3.3	<0.001
platelet (×10^3^/µL)	21.7 ± 5.0/22.4 ± 5.3	0.274
HbA1c (%)	5.7 ± 0.6/5.6 ± 0.4	0.142
WBC (×10^3^/µL)	6.53 ± 1.77/5.86 ± 1.38	<0.001
Total protein (g/dL)	7.39 ± 0.34/7.45 ± 0.37	0.125
Total cholesterol (mg/dL)	205.4 ± 33.2/213.6 ± 29.1	0.020
Triglycerides (mg/dL)	109.3 ± 63.7/88.9 ± 50.2	0.002
HDL (mg/dL)	58.5 ± 12.7/66.8 ± 14.7	<0.001
LDL (mg/dL)	121.9 ± 30.5/123.5 ± 26.4	0.615
Serum creatinine (mg/dL)	0.825 ± 0.134/0.657 ± 0.109	<0.001
BMI (kg/m^2^)	24.7 ± 3.2/23.6 ± 6.2	0.006

**Figure 1:**
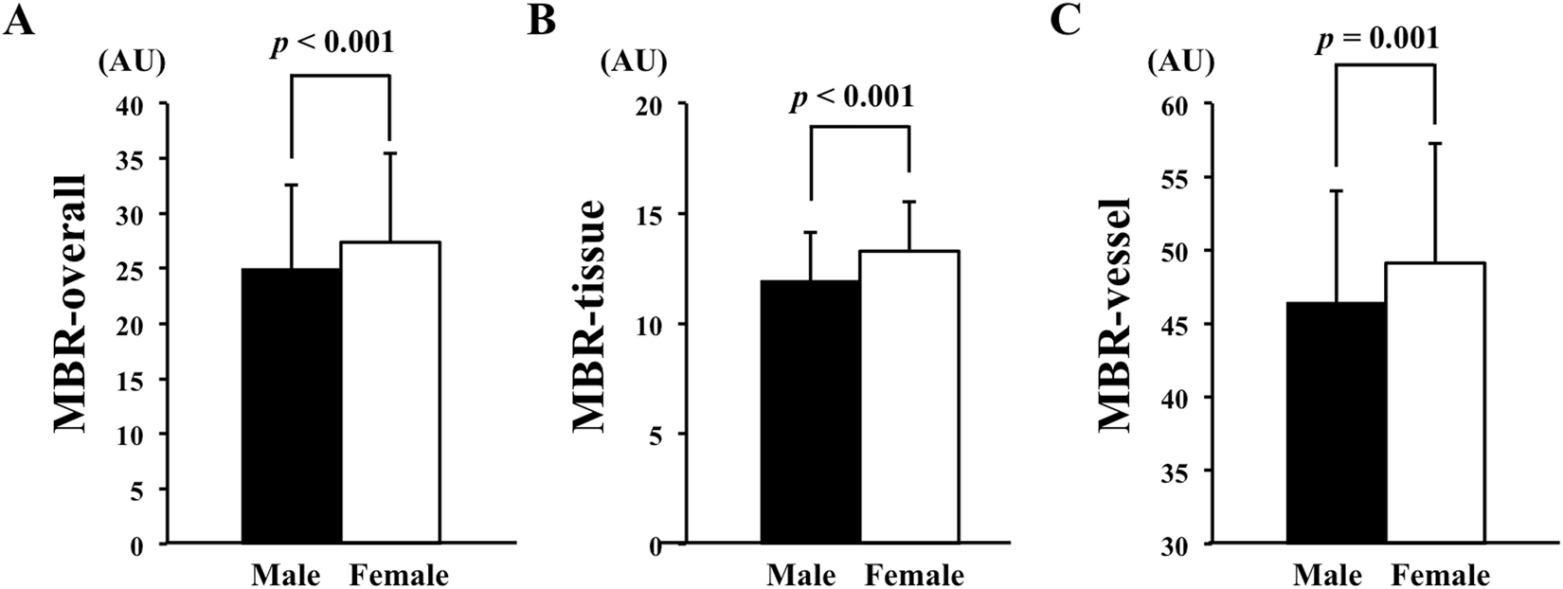
Differences between the sexes in the MBR determined by LSFG. The MBR of the ONH in the female group was significantly higher than that in the male group for the over-all ONH (*P* < 0.001) (**A**), the tissue (*P* < 0.001) (**B**), and the vessels (*P* = 0.001) (**C**).

The MBR-overall (*P* < 0.001), the MBR-tissue (*P* < 0.001), and the MBR-vessel (*P* = 0.001) of the ONH were significantly higher in the female group than that in the male group (Fig. 1).

**Figure 2:**
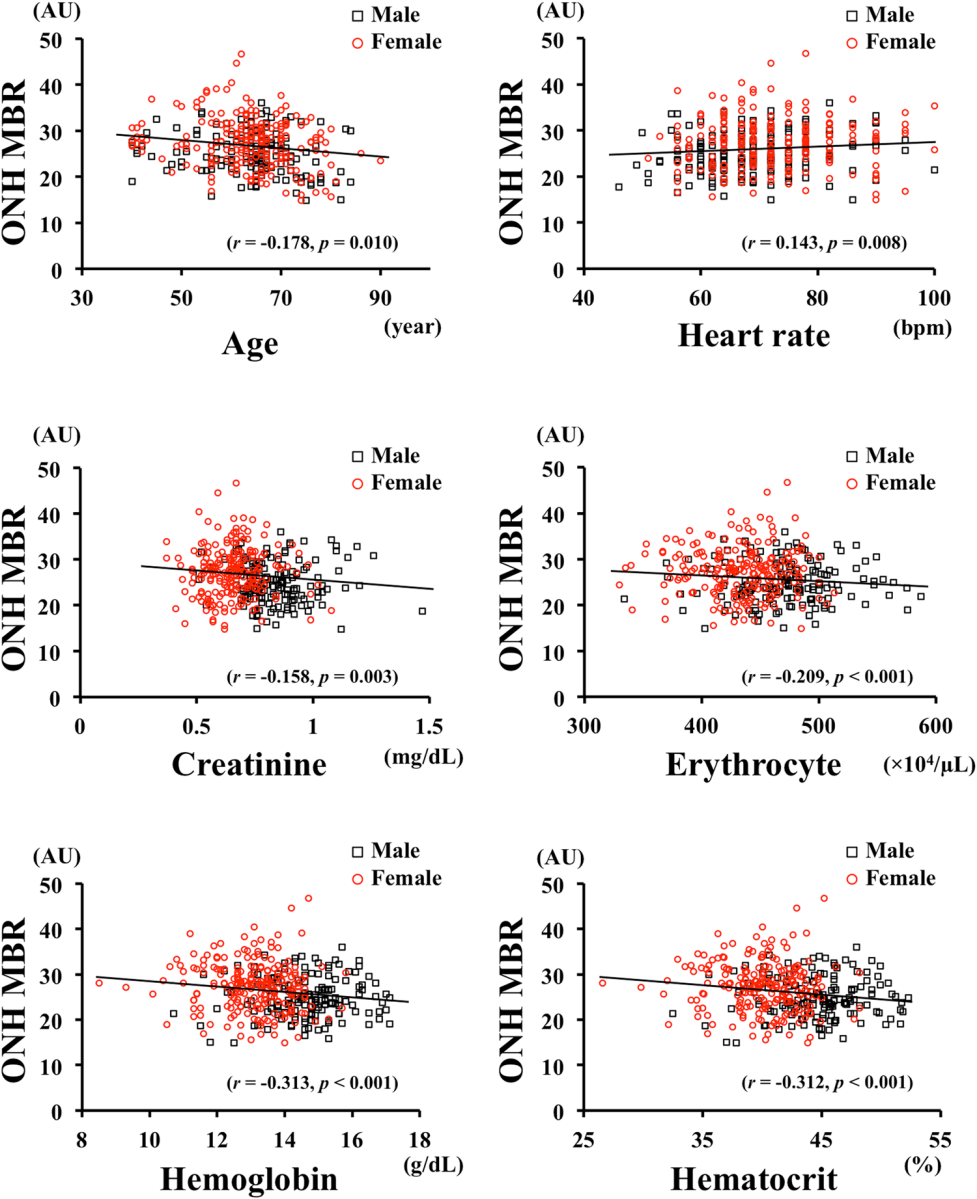
Correlation between the ONH-MBR and the biological characteristics of the subjects. The MBR was significantly correlated with age (*r* = −0.178, *P* = 0.010), heart rate (*r* = 0.143, *P* = 0.008), serum creatinine (*r* = −0.158, *P* = 0.003), erythrocyte (*r* = −0.209, *P* < 0.001), hemoglobin (*r* = −0.313, *P* < 0.001), and hematocrit (*r* = −0.312, *P* < 0.001). Black square, males; Red circle, females.

**Figure 3:**
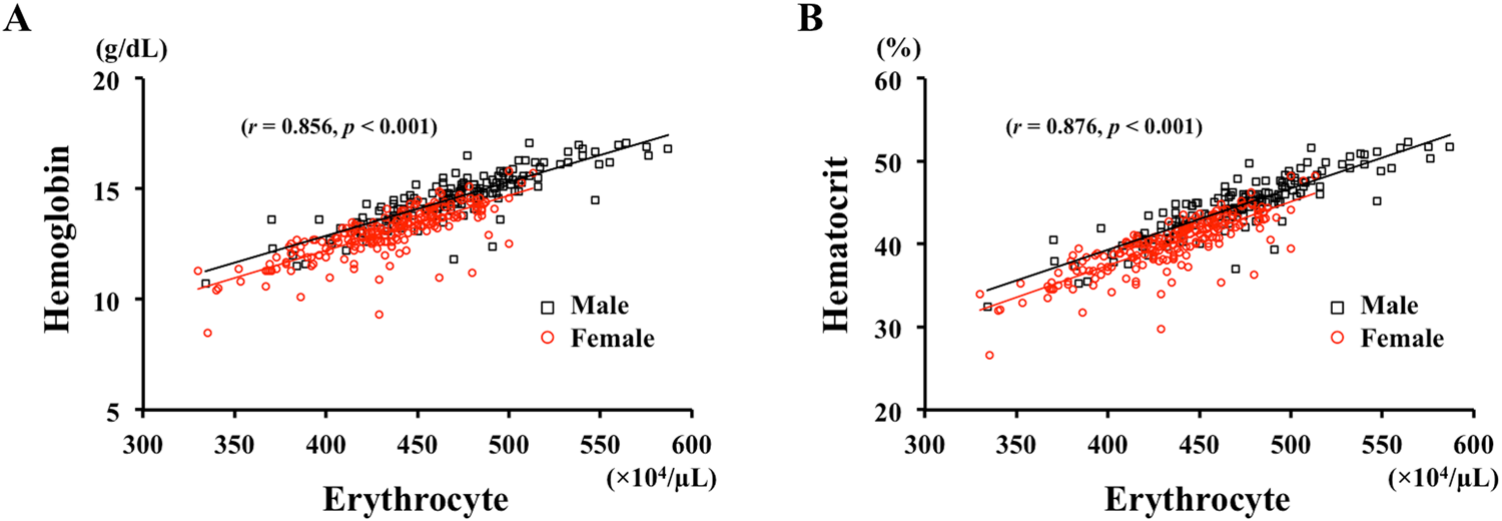
Correlation between the erythrocyte concentration and the hemoglobin concentration and the hematocrit percentage. The erythrocyte concentration was significantly and positively correlated with the hemoglobin concentration (*r* = 0.856, *P* < 0.001) (**A**) and hematocrit percentage (*r* = 876, *P* < 0.001) (**B**).

The results of the Spearman’s coefficients of correlation are shown in Table 2. The MBR-overall (*r* = 0.254, *P* < 0.001), MBR-tissue (*r* = 0.303, *P* < 0.001), and MBR-vessel (*r* = 0.163, *P* = 0.001) were significantly correlated with the sex. The MBR-overall, MBR-tissue, and MBR-vessel of the ONH were not significantly different, and only the MBR-overall on the ONH was used for further analyses. The MBR-overall was correlated with age (*r* = −0.178, *P* = 0.010), heart rate (*r* = 0.143, *P* = 0.008), erythrocyte (*r* = −0.209, *P* < 0.001), hemoglobin (*r* = −0.313, *P* < 0.001), hematocrit (*r* = −0.312, *P* < 0.001), and serum creatinine (*r* = −0.158, *P* = 0.003; Fig. 2). The erythrocyte was positively correlated with the hemoglobin concentration (*r* = 0.856, *P* < 0.001) and the hematocrit percentage (*r* = 0.876, *P* < 0.001; Fig. 3).

**Table 2:** Result of Spearman’s rank correlation coefficient between the MBRs and clinical parameters.

Parameters		Gender	Age	IOP	AL	SBP	DBP	MOPP	HR	RBC	Hb	
**MBR**												
ONH	overall	0.254^a^	−0.178^b^	−0.021	−0.102	−0.092	−0.141^c^	−0.031	0.143^b^	−0.209^a^	−0.313^a^	
	tissue	0.303^a^	−0.003	−0.025	−0.151^b^	−0.115^c^	−0.178^b^	−0.051	0.153	−0.220^a^	−0.264^a^	
	vessel	0.163^b^	−0.137^c^	−0.063	−0.035	−0.119^c^	−0.129^c^	−0.025	0.078	−0.129^c^	−0.211^a^	
**Parameters**		**Ht**	**Plt**	**HbA1c**	**WBC**	**TP**	**TG**	**Tchol**	**HDL**	**LDL**	**Cre**	**BMI**
**MBR**												
ONH	overall	−0.312^a^	−0.069	−0.060	0.038	0.052	0.018	0.052	−0.027	0.058	−0.158^b^	0.009
	tissue	−0.261^a^	−0.083	0.006	−0.050	0.070	−0.065	0.106	0.091	0.064	−0.130^c^	−0.085
	vessel	−0.218^a^	0.076	−0.035	0.048	−0.010	0.029	0.088	−0.040	0.100	−0.099	0.034

MBR: mean blur rate; ONH: optic nerve head; IOP: intraocular pressure; AL: axial length; SBP: systolic blood pressure; DBP: diastolic blood pressure; MOPP: mean ocular perfusion pressure; HR: heart rate; RBC: retinal blood cell; Hb: hemoglobin; Ht: hematocrit; Plt: platelet; WBC: white blood cell; TP: total protein; TG: triglycerid; Chol: cholesterol; HDL: high-density lipoprotein; LDL: low- density lipoprotein; Cre: creatinine; BMI: Body mass index, ^a^ *p* < 0.001, ^b^ *p* < 0.01, ^c^ *p* < 0.05.

Multiple stepwise regression analyses showed that the sex (*β* = 0.248, *P* < 0.001), age (*β* = −0.185, *P* < 0.001), and axial length (*β* = −0.122, *P* = 0.027) were factors independently correlated with the ONH-MBR when the clinical examination data were not included in the analysis (Table 3). However, hemoglobin (*β* = −0.295, *P* < 0.001), age (*β* = −0.264, *P* < 0.001), and axial length (*β* = −0.150, *P* = 0.006) were independent factors contributing to the ONH-MBR when the clinical examination data were included (Table 4). For each sex, the hemoglobin (*β* = −0.225, *P* = 0.011), age (*β* = −0.222, *P* = 0.012), and heart rate (*β* = 0.206, *P* = 0.014) in the males (Table 5) and age (*β* = −0.286, *P* < 0.001), hemoglobin (*β* = −0.217, *P* = 0.002), and axial length (*β* = −0.173, *P* = 0.023) in the females (Table 6) were factors contributing independently to the ONH-MBR when the clinical examination data were included.

**Table 3:** Result of multiple stepwise regression analysis for independence of factors not including laboratory data contributing to ONH MBR.

Dependent	Independent	*β*	*P*-value
Variable			
ONH MBR	Gender	0.248	<0.001
	Age	−0.185	<0.001
	Axial length	−0.122	0.027
	Heart rate	0.079	0.152
	MOPP	−0.022	0.685

ONH: optic nerve head; MBR: mean blur rate; MOPP: mean ocular perfusion pressure.

**Table 4:** Result of multiple stepwise regression analysis for independence of factors including laboratory data contributing to ONH MBR.

Dependent	Independent	*β*	*P*-value
Variable			
ONH MBR	Hemoglobin	−0.295	<0.001
	Age	−0.264	<0.001
	Axial length	−0.150	0.006
	Heart rate	0.102	0.090
	Gender	0.262	0.062
	MOPP	0.728	−0.019
	Creatinine	0.742	0.018

ONH: optic nerve head; MBR: mean blur rate; MOPP: mean ocular perfusion pressure.

**Table 5:** Result of multiple stepwise regression analysis for independence of factors including laboratory data contributing to ONH MBR in the male group.

Dependent	Independent	*β*	*P*-value
Variable			
ONH MBR	Hemoglobin	−0.225	0.011
	Age	−0.222	0.012
	Heart rate	0.206	0.014
	MOPP	0.116	0.180
	Axial length	−0.080	0.359
	Creatinine	0.010	0.911

ONH: optic nerve head; MBR: mean blur rate; MOPP: mean ocular perfusion pressure.

**Table 6:** Result of multiple stepwise regression analysis for independence of factors including laboratory data contributing to ONH MBR in the female group.

Dependent	Independent	*β*	*P*-value
Variable			
ONH MBR	Age	−0.286	<0.001
	Hemoglobin	−0.217	0.002
	Axial length	−0.173	0.023
	Creatinine	0.109	0.134
	MOPP	−0.073	0.319
	Heart rate	0.010	0.889

ONH: optic nerve head; MBR: mean blur rate; MOPP: mean ocular perfusion pressure.

## Discussion

Single linear regression analysis showed that the ONH-MBR was significantly correlated with the sex, and the ONH-MBR was significantly higher in the female group than that in the male group. In addition, some of the clinical examination data, e.g., erythrocyte, hemoglobin, and hematocrit, were significantly different between the sexes. The ONH-MBR was significantly and negatively correlated with the erythrocyte count, hemoglobin concentration, hematocrit percentage, and other clinical examination data. The multiple stepwise regression analyses showed that sex was an independent factor correlated with the ONH-MBR when the clinical examination data were not included in the analysis. On the other hand, sex was not and hemoglobin was an independent factor correlated with the ONH-MBR when clinical examination data were included in both the male and female groups.

It was recently reported that the sex-related differences were present in ocular blood flow in the ONH^25,26^. Our result showed sex-related differences of the ONH-MBR which was faster in the female group than that in the male group which agrees with earlier studies^25,26^. In the brain, males have lower cerebral blood flow velocity than females up to the age of 80 years^27^. Vavilala *et al*. reported that females had higher blood flow velocities in both the basilar and the common carotid arteries^28^. These results showed that the blood flow velocity is faster in females not only in the ONH but also in the other organs. Thus, the sex-related differences in the blood flow may be a systemic phenomenon.

LSFG measures the ocular blood flow by examining the pattern of the speckle contrast produced by the movement of the erythrocytes in the ocular blood vessels. Accordingly, the erythrocyte level should affect the results of ocular blood flow determined by LSFG. In addition, the erythrocyte level is linearly correlated with the hemoglobin and the hematocrit levels, and an increase in the number of erythrocytes causes both an increase in the hemoglobin concentration and the hematocrit percentage in the healthy subjects.

For normal retinal and ONH function, an optimal regulation of the blood flow in the ONH is necessary to maintain a constant oxygen supply. The hemoglobin concentration is related to the oxygen carrying capacity of blood, and the arterial oxygen content is determined by the hemoglobin concentration. This is then an indicator of the arterial oxygen content^29,30^. Low arterial oxygen levels will increase the blood flow by vasodilation to maintain an adequate supply of oxygen^31^. The retinal blood flow increases in response to a reduction in oxygen (hypoxia) and decreases in response to increased oxygen (hyperoxia)^32^. Females have a lower blood oxygen capacity than males because of a lower number of erythrocytes that contain hemoglobin.

The blood viscosity is directly related to the hematocrit percentage, and an increase in the hematocrit percentage leads to an increase in the relative viscosity^33^. An increase in the viscosity such as that caused by a reduction of the water content of the serum can lead to increases in the resistance of blood flow and thus a reduction in flow. Consequently, these changes can cause a higher hematocrit percentage that can then cause lower blood flow velocity. The levels of hemoglobin and hematocrit are important determinants of the arterial oxygen content and viscosity of the blood and can therefore influence ocular blood flow. Thus, we investigated the relationship between the levels of hemoglobin or hematocrit and the ONH-MBR to determine the mechanism of the sex-related differences in the ocular blood flow. These hematological parameters, e.g. erythrocytes, hemoglobin, and hematocrit, were very highly correlated with each other, and we examined only the hemoglobin for the independent factor in the multiple regression analyses to avoid multicollinearity.

There have been several reports describing the relationship between the blood flow velocity and hemoglobin or hematocrit levels. Earlier studies showed that the blood flow velocity was negatively correlated with the hemoglobin concentration and hematocrit levels^34^. The blood flow velocity in the greater middle cerebral artery velocity in females may be in part associated with their lower hematocrit level which is consistent with the inverse correlation between the cerebral blood flow and the hematocrit level. In the healthy brain, the cerebral blood flow velocity increases with a reduction in the blood viscosity and arterial oxygen concentration^35^. In addition, studies in patients with abnormally high or low hematocrit levels have shown that normalization of the hematocrit level leads to cerebral blood flow values to change to be within the normal range^29,36^. The arterial oxygen content is determined by both the hematocrit and hemoglobin levels, and higher levels of cerebral blood flow have been observed in anemic patients than healthy controls^30^. In addition, females have the possibility that the supply of oxygen will change because the hemoglobin and hematocrit levels change even in a month because of menstruation. Taken together, the negative correlation between the hemoglobin or hematocrit levels and blood flow velocity is observed not only on the ONH but also in other organs and should be an optimal systemic regulation.

**Figure 4:**
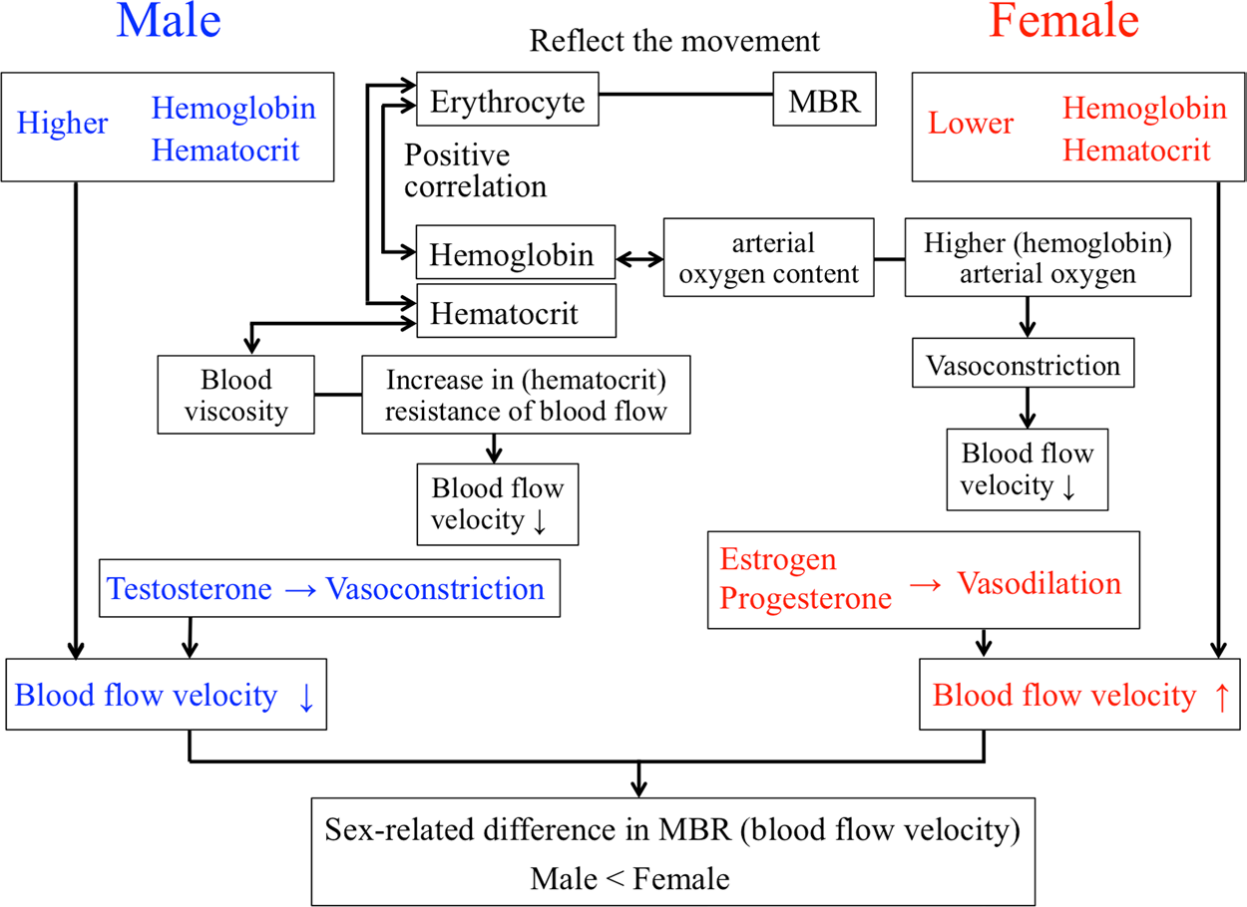
A schematic diagram to present the hypothesis.

The results showed that the male group had higher hemoglobin and hematocrit levels and lower ONH blood flow velocity, and the female group had lower hemoglobin and hematocrit and higher ONH blood flow velocity. The multiple regression study showed that sex-related differences were present when the clinical examination data were not included which is consistent with the results of earlier results^25,26^. However, the multiple stepwise regression analyses showed that sex was not an independent factor for the ONH-MBR blood flow when clinical examination data were included in the analyses. In addition, the hemoglobin level contributed to the ONH-MBR in both the male and female groups. These results indicated that the sex-related differences in the hemoglobin and hematocrit levels, and the negative correlations between the hemoglobin and hematocrit levels and the ONH-MBR are related to the cause the sex-related differences in the ONH-MBR (Fig. 4).

The results of earlier studies suggested that the hormonal status and biological characteristics^22,25^ to be other possibilities for the sex-related differences in blood flow in the ONH. There are several reports that the sex hormone level in the serum was related to the blood flow velocities and resistive indices^21,23,24^. Estrogen and progesterone promote vasodilation^21^ while testosterone causes vasoconstriction through the induction of thromboxane production^21^. Rodriguez *et al*. found a significantly higher cerebral blood flow in premenopausal females than age-matched males^37^. Harris–Yitzhak *et al*. suggested that the extrabulbar branches of the ophthalmic artery may be responsible for this decrease in vascular resistance caused by estrogen^38^. These results suggested that the sex hormones can affect the ocular blood flow and cause the sex-related differences.

The multiple regression analyses showed that age was an independent factor contributing the ONH-MBR in both groups. In addition to our results, there have been many reports describing the negative correlation between the age and blood flow in the ONH^18,39–42^. In addition, the retrobulbar vessels and retinal perfusion have also been shown to decrease with increasing age^43^. Aging leads to arteriosclerosis and a reduction of the elasticity of the fibers causes a hardening of the arterial wall. However, there is no significant difference in aging between the male and the female group, thus it is less likely that age is a contributing factor for the sex-related differences in the ocular blood flow.

The sex-related differences in the ocular blood flow could be related to the lower body height and size, the higher heart rates, and lower cardiac outputs in females^22^. The shorter stature places the site of arterial pulse reflection closer to the heart which brings the reflected wave back to the central aorta earlier in systole with a resultant decrease in the pulse amplification. Our result showed that the BMI was not a factor contributing to the sex-related differences in the ocular blood flow. Therefore, it is less likely that the biological characteristics are factor contributing to the sex-related differences in the ocular blood flow.

The SBP, DBP, and MOPP were significantly lower in females than males, but the MBR-ONH was significantly higher in females than males. A lower MOPP should cause a lower ocular blood flow. The lower blood viscosity in females associated with the lower hematocrit level should decrease the mechanical energy less for the blood flow against viscous resistances^44^, and erythrocytes probably move faster in the ocular vessels in females in the ONH although the MOPP was not higher.

The axial length is longer in males than females in our cohort. It has been reported that the axial length is negatively correlated with the ocular blood flow^45–48^. Shimada *et al*. reported that the rate of retinal blood flow was lower in eyes with high myopia, mainly due to the narrowing of the diameter of retinal vessels^47^. On the other hand, it had been reported that there is no significant correlation between the rate of retinal blood flow and the axial length using Doppler Fourier-domain optical coherence tomography scans^49^. The single regression analysis indicated that the MBR was not significantly correlated with the axial length. However, it is not possible to eliminate the idea that the axial length may have affected the ONH MBR, i.e. a longer axial length in females leads to a higher MBR as a potential bias.

The specific features of the blood flow in females such as a faster erythrocyte flow with lower hemoglobin and hematocrit levels, and the specific hormonal status should be related to the lower number of cardiovascular events in females^50–52^. There is evidence that thromboembolic events happen more frequently in males than females. A large longitudinal study carried out in the US found that the incidence of central retinal vein occlusion was higher in males than in females^53,54^. A Korean nationwide epidemiologic study of central retinal artery occlusion showed that the incidence was 1.47 times higher in males than in females^55^. In addition, the incidence of retinal emboli was higher in males than in females^56^.

Our study has limitations. First, the level of sex hormones was not measured even though they may play a role in the sex-related differences in the ocular blood flow. Second, we did not determine the menopausal status of the females, and thus we could not compare the ocular blood flow between pre- and post-menopausal females. Third, the age of all of the volunteers was ≥40 years meaning that younger individuals were not studied. It has been reported that the intercept of the regression line of the MBR and age in the older age group was steeper than that in the younger age group (<45 years) in the female group, and that the intercepts of the regression lines of the MBR in the older male and female groups were very similar^25^. Accordingly, if females younger 40 years had been included, the results may have been different. Further studies with the sex hormonal data, e.g., estrogen and testosterone levels, from a larger number and wider range of healthy subjects will be necessary to determine the mechanism for the sex-related differences in the ocular blood flow.

In conclusion, the results showed that the factors causing the sex-related differences in the blood flow on the ONH are the differences in the hemoglobin and hematocrit levels and negative correlation between the hemoglobin and hematocrit levels. We believe that our findings of the sex-related differences in ocular blood flow needs to be considered when interpreting blood flow data not only in the ocular system but also the systemic system.

## Methods

### Subjects and testing protocol

This was a prospective study, and the procedures used were approved by the Ethics Committee of Nagoya University Hospital. The procedures conformed to the tenets of the Declaration of Helsinki, and a written informed consent was obtained from each subject after an explanation of the nature and possible complications of the procedures.

The subjects were ≥40 years who attended a basic health checkup in 2015 supported by a local government. This checkup, the Yakumo study, was conducted in the town of Yakumo in a rural area of southern Hokkaido, Japan. All of the subjects had a best-corrected visual acuity (BCVA) of ≥20/30. Evidence of an absence of severe cataract, glaucoma, or chorioretinal diseases were determined by slit-lamp examination, OCT, and color photography. Subjects were also screened for any medical conditions that could influence the hemodynamics of the eye such as diabetes, hypertension, arrhythmia, and vascular diseases. Also, individuals who had had ocular laser treatment of the experimental eye, were taking topical or systemic medications including hormonal medications, or had axial lengths >26.5 mm^47^ were excluded.

Only one randomly-selected eye/volunteer was used for the measurements. All participants were asked to abstain from alcoholic and caffeinated beverages on the morning of the day of the examination because the intake of alcohol and caffeine can influence the intraocular pressure (IOP)^57,58^ and blood pressure^59,60^. The subjects rested for 10 to 15 min in a quiet dark room before the measurements. All examinations were performed in the sitting position.

The axial lengths were measured by partial optical coherence interferometry (IOLMaster; Carl Zeiss Meditec, La Jolla, CA). The IOP was measured with a handheld tonometer (Icare; Tiolat Oy, Helsinki, Finland). The SBP and the DBP were measured at the left brachial artery at the height of the heart in a sitting position with an automatic sphygmomanometer (CH-483C; Citizen, Tokyo, Japan). The MAP and the MOPP were calculated as: MAP = DBP + 1/3(SBP-DBP) and MOPP = 2/3MAP – IOP^61^.

For the hematological analyses, the erythrocyte (×10^4^/µL), hemoglobin (g/dL) hematocrit (%), platelet (×10^3^/µL), HbA1c (%), white blood cell (×10^3^/µL), total protein (g/dL), triglycerides (mg/dL), total cholesterol (mg/dL), HDL (mg/dL), LDL (mg/dL), serum creatinine (mg/dL) were examined. The bodyweight and height were measured, and the BMI was calculated as: BMI = bodyweight in kg/height in meter^2^.

### Laser Speckle Flowgraphy

**Figure 5:**
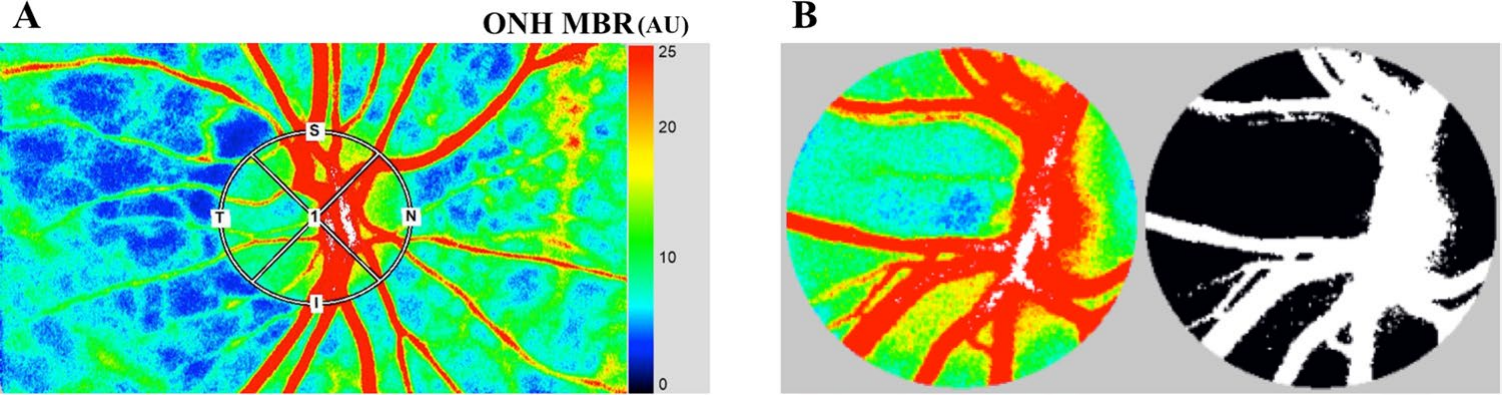
Representative composite color maps used to determine the mean blur rate (MBR) recorded by laser speckle flowgraphy (LSFG). The red color indicates a high MBR and the blue color indicates a low MBR. To measure the MBR on the optic nerve head (ONH), a circle was drawn around the ONH (**A**). A binary format image for segmentation between the vessel (white area) and tissue (black area) areas on the ONH (**B**).

The principles of LSFG have been described in detail^62–65^. Briefly, this instrument consists of a fundus camera equipped with an 830 nm diode laser light source and a standard charge-coupled sensor (750 width × 360 height pixels) as the detector. After switching on the laser, a speckle pattern appears due to the interference of the light scattered by the movements of the erythrocytes. The MBR is a measure of the relative blood flow velocity, and it is determined by examining the pattern of the speckle contrast produced by the erythrocytes in the blood vessels. The MBR images are acquired at a rate of 30 frames/s over a 4-s period. To evaluate the ONH, a circle is set surrounding the ONH (Fig. 5A). The “vessel extraction” function of the software then identifies the vessel and tissue areas on the ONH so that the MBR could be assessed separately (Fig. 5B). The MBR was determined in three areas: the overall, the vessels, and the tissue areas on the ONH. The software in the instrument is able to track the eye movements during the measurement period. The LSFG was measured two times at each time point. The average of the variables derived with LSFG was calculated.

### Statistical Analyses

The values of each parameter are presented as the means ± standard deviations. Independent *t* tests were used to compare normally distributed data. Spearman’s rank test was used to determine the correlation coefficients between the variables. Simple regression analyses were used to investigate the correlation among the erythrocytes, hemoglobin, and hematocrit. Stepwise multiple regression analyses were used to determine independent factors affecting the ONH-MBR. All statistical analyses were performed using IBM SPSS Statistics for Windows, Version 24.0 (IBM Corp., Armonk, NY). The significance level was set at *P* < 0.05.
